# Safety and effectiveness of the SUPRACOR presbyopic LASIK algorithm on hyperopic patients

**DOI:** 10.1186/s40662-016-0062-6

**Published:** 2016-12-08

**Authors:** Robert Edward T. Ang, Emerson M. Cruz, Alex U. Pisig, Maria Luisa Patricia C. Solis, Rosalie Mae M. Reyes, Gerhard Youssefi

**Affiliations:** 1 Asian Eye Institute, 8th Floor PHINMA Plaza, Rockwell Center, Makati City, 1200 Philippines; 2 Department of Ophthalmology, Cardinal Santos Medical Center, 10 Wilson St. Greenhills West, San Juan City, Philippines; 3 TECHNOLAS PerfectVision GmbH, A Bausch & Lomb Company, Messerschmittstrasse 1-3, Munich, 80992 Germany

**Keywords:** Presbyopia, LASIK, Supracor, Hyperopia

## Abstract

**Background:**

To evaluate the safety and effectiveness of the Supracor excimer laser algorithm to treat hyperopic presbyopic patients using laser in-situ keratomileusis (LASIK).

**Methods:**

This is a retrospective case review of patients diagnosed with hyperopia (Sphere ≥ +0.0 D and presbyopia reading add ≥ 1.0 D) who underwent Supracor excimer laser treatment on at least one eye for presbyopia correction from year May 2011 to May 2013. Binocular vision was further analyzed after patients were subdivided into three groups: Group A (*n* = 22 eyes, 11 patients) had Supracor on both eyes; Group B (*n* = 18 eyes, 18 patients) had Supracor in one eye and hyperopic LASIK on fellow eye; and Group C (*n* = 29 eyes, 29 patients) had Supracor in one eye and no treatment on the fellow eye.

**Results:**

This study evaluated 58 patients wherein 69 eyes underwent Supracor presbyopic LASIK. Preoperatively, mean manifest refraction spherical equivalent (MRSE) of all eyes that underwent Supracor was +1.37 ± 0.72 D with mean uncorrected distance visual acuity (UDVA), uncorrected intermediate visual acuity (UIVA), and uncorrected near visual acuity (UNVA) of 20/50 (0.35 logMAR), 20/50 (0.35 logMAR), and J9 (0.61 logMAR), respectively. At 6 months postoperatively, mean MRSE was −0.43 ± 0.59 D with mean UDVA, UIVA and UNVA of 20/25 (0.13 logMAR), 20/20 (0.01 logMAR), and J1 (0.05 logMAR), respectively. Loss of two lines of best-corrected distance visual acuity (BCDVA) was seen in 6% of eyes. Mean corneal steepening of 1.0 D at the 3 mm zone and 0.7 D in the 5 mm zone was observed. Mean vertical coma increased from −0.02 to +0.10 while mean 4th order spherical aberration became more negative from 0.20 to −0.14. Mean binocular UDVA, UIVA, and UNVA are 20/20, 20/20 and J1, respectively, in all treatment groups at the 6 month postoperative follow-up. No significant differences in binocular UDVA (p ≥ 0.36), UIVA (p ≥ 0.19) and UNVA (p ≥ 0.56) among groups were seen.

**Conclusions:**

Supracor excimer laser algorithm is safe and effective for the treatment of presbyopia in hyperopes. Monolateral and bilateral Supracor treatments yielded similarly good binocular vision outcomes.

## Background

Presbyopia is a condition wherein the ability to read small print weakens as one ages beyond 40 years old. Non-surgical solutions are reading glasses or contact lenses. Surgical solutions include cornea-based treatments such as laser vision correction or inlays while lens-based solutions include multifocal or accommodating intraocular lenses implanted after removal of the natural lens.

Laser in-situ keratomileusis (LASIK) involves creating a flap and reshaping the cornea to correct refractive power and improve distance vision. LASIK is also being used to correct presbyopia in a variety of ways. In conventional monovision LASIK, the dominant eye is targeted for Plano refraction for good distance vision while the non-dominant eye is targeted to −1.50 D for good near vision [1, 2]. More advanced laser algorithms have been developed to produce a multifocal ablation or manipulate asphericity to improve near vision [3–9].

Multifocal ablations can be categorized into “center-distance” or “center-near” ablation profiles. The Pseudo accommodative Cornea (PAC, Nidek, Aichi, Japan) creates an aspheric cornea that is flatter in the center to provide good distance vision (Center-distance) and is steeper away from the center forming a peripheral near zone (concentric ring for near vision) [10]. The Supracor (Bausch and Lomb Technolas, Munich, Germany) and PresbyMax (Schwind, Kleinostheim, Germany) algorithms create topographic profiles wherein there is an elevation in the center of the cornea for good reading vision (Center-near) and flatter topography towards the periphery for good intermediate and distance vision [11, 12].

While presentations in congresses and publications report good outcomes in terms of improved near vision, it is difficult to compare the ablations directly with each other because the algorithms are proprietary and specific to a particular brand of laser equipment. In addition, the laser treatment algorithms across different brands are at different stages of product development. Therefore, comparisons may not be equal if the algorithm is not in its final commercial form.

Supracor creates a varifocal cornea wherein there is a 12 μm elevation in the central 3 mm of the cornea to give a near addition of approximately two diopters (D). Outside of the near addition is an aberration-optimized transition zone that gives good intermediate and good distance vision. The algorithm is available in the Technolas 217P and Teneo 317 excimer lasers (Bausch and Lomb Technolas, Munich, Germany). Since Supracor is a LASIK-based algorithm, its main advantage is it can correct refractive error and presbyopia in a single procedure. In clinical practice, Supracor can be used in one eye or in both eyes depending on each patient’s needs and expectations.

The objective of our retrospective study is to evaluate the safety and effectiveness of the SUPRACOR presbyopic excimer laser treatment algorithm for hyperopic eyes, with or without astigmatism. A secondary objective is to compare the binocular visual outcomes of hyperopic patients that underwent Supracor in one or both eyes.

## Methods

This is a retrospective, single center, single surgeon, case series of hyperopic presbyopic patients who underwent presbyopic Supracor LASIK treatment. This study was performed according to the tenets of the Declaration of Helsinki and was approved by the ethics committee of our institution. In Supracor-treated eyes, a 120 μm flap was created using either the XP microkeratome (Bausch and Lomb Technolas, Munich, Germany) or the Victus femtosecond laser (Bausch and Lomb Technolas, Munich, Germany). The Technolas 217P laser (Bausch and Lomb Technolas, Munich, Germany) was used to perform excimer laser treatment. The recommended refractive target for Supracor was −0.50 D spherical equivalent (SE). The optical zone size was 6 mm. Postoperative topical medication regimen consisted of Levofloxacin (Oftaquix, Santen Pharmaceutical, Osaka, Japan) four times a day, Prednisolone acetate 1% (Pred forte, Allergan, California, USA) every hour for 2 days then tapered to four times a day, and Ketorolac (Acular, Allergan, California, USA) four times a day.

### Outcomes

The data of all patients diagnosed with hyperopia (Sphere ≥ +0.0 D) and presbyopia (reading add ≥ 1.0 D) who underwent SUPRACOR presbyopic excimer laser treatment on at least one eye for near indication from May 2011 to May 2013 with a minimum follow-up of 1 month were retrospectively reviewed. The data of 69 Supracor-treated eyes from 58 patients were included in this study. Excluded were the presence of ocular surface disease, abnormal corneal topography, and less than 5.0 mm wetting on Schirmer testing over 5 min without topical anesthesia.

Patient demographics, manifest refraction, eye dominance, monocular and binocular distance, intermediate and near visual acuity measurements were obtained on the pre-operative, 1 week, 1 month, 3 months, and 6 months post-operative follow-up. Corneal topography using the Orbscan IIz (Bausch & Lomb, Munich, *Germany*), and undilated and dilated wavefront aberrometry measurements using the Zywave II (Bausch & Lomb, Munich, *Germany*) were likewise obtained for this study on the pre-operative, 1 month, 3 months, and 6 months post-operative follow-up.

Supracor treatment outcomes were analyzed based on effectiveness: uncorrected distance visual acuity (UDVA), uncorrected intermediate visual acuity (UIVA), uncorrected near visual acuity (UNVA), manifest refraction spherical equivalent (MRSE), and safety (loss of lines of best corrected distance visual acuity)–change in corneal topography and change in the 6 mm higher order aberration from preoperative to 6 month postoperative visit. A sub-analysis of binocular visual outcomes was performed after patients were subdivided into three groups: Group A: bilateral Supracor; Group B: one eye Supracor, fellow eye treated with hyperopic LASIK; Group C: one eye Supracor, fellow eye untreated.

### Statistical analysis

Data were encoded and tallied in Microsoft Excel 2007 and descriptive statistics were generated for all variables. Visual acuity was expressed in the logarithm of minimum angle of resolution (LogMAR) scale for the analysis. For numerical data, mean and standard deviation were generated. One way Analysis of Variance, and paired two-tailed *T*-test on samples were performed for the quantitative data with the significance level set at *p* < 0.05.

## Results

From May 2011 to May 2013, 69 eyes of 58 hyperopic patients, consisting of 25 males and 33 females, underwent Supracor presbyopic laser treatment for near indication. The mean age was 51.6 years. Analysis of patients was further subdivided to three treatment groups: Group A (*n* = 11 patients) had bilateral Supracor treatment; Group B (*n* = 18 patients) had Supracor on one eye and Hyperopic LASIK on the fellow eye; and Group C (*n* = 29 patients) underwent Supracor on one eye and no treatment on the fellow eye (Table 1).

**Table 1:** Demographics

	Supracor + Supracor	Supracor + Standard LASIK	Supracor + No Treatment	*P* Value
Patients	11	18	29	n/a
Male	3	10	12	n/a
Female	8	8	17	
Mean Age (Years)	53.18 ± 3.20	50.56 ± 4.38	50.83 ± 4.68	0.70
UDVA	0.63 ± 0.18	0.43 ± 0.21	0.05 ± 0.06	0.00
UIVA	0.40 ± 0.00	0.40 ± 0.00	0.30 ± 0.12	0.16
UNVA	0.70 ± 0.00	0.68 ± 0.06	0.53 ± 0.22	0.16
Sphere (D)	1.98 ± 0.39	1.97 ± 0.69	1.02 ± 0.52	0.00
Cylinder (D)	−0.39 ± 0.26	−0.33 ± 0.33	−0.47 ± 0.49	0.46
Spherical Equivalent (SE) (D)	1.78 ± 0.34	1.81 ± 0.72	0.78 ± 0.48	0.00

*UDVA*= uncorrected distance visual acuity; *UIVA*= uncorrected intermediate visual acuity; *UNVA*= uncorrected near visual acuity

## Supracor-treated eyes

### Efficacy

Pre-operatively, the mean MRSE of all Supracor-treated eyes was 1.37 ± 0.72 D, mean sphere was +1.57 ± 0.71 D and mean astigmatism was −0.41 ± 0.39 D. At 6 months postoperatively, the mean MRSE was −0.43 ± 0.59 D, the mean sphere was −0.20 ± 0.57 D and the mean astigmatism was −0.47 ± 0.29 D (Table 2).

**Table 2:** Refractive Outcome

Time (*N* = eyes)	Sphere (D)		Cylinder (D)		Spherical Equivalent (MRSE) (D)	
	Mean	St-Dev	Mean	St-Dev	Mean	St-Dev
Pre-Op (69)	1.57	0.71	−0.41	0.39	1.37	0.72
1 Week (62)	−0.37	0.56	−0.58	0.32	−0.66	0.53
1 Month (64)	−0.42	0.68	−0.64	0.36	−0.74	0.67
3 Months (45)	−0.26	0.58	−0.59	0.29	−0.55	0.55
6 Months (38)	−0.20	0.57	−0.47	0.29	−0.43	0.59

**Fig. 1:**
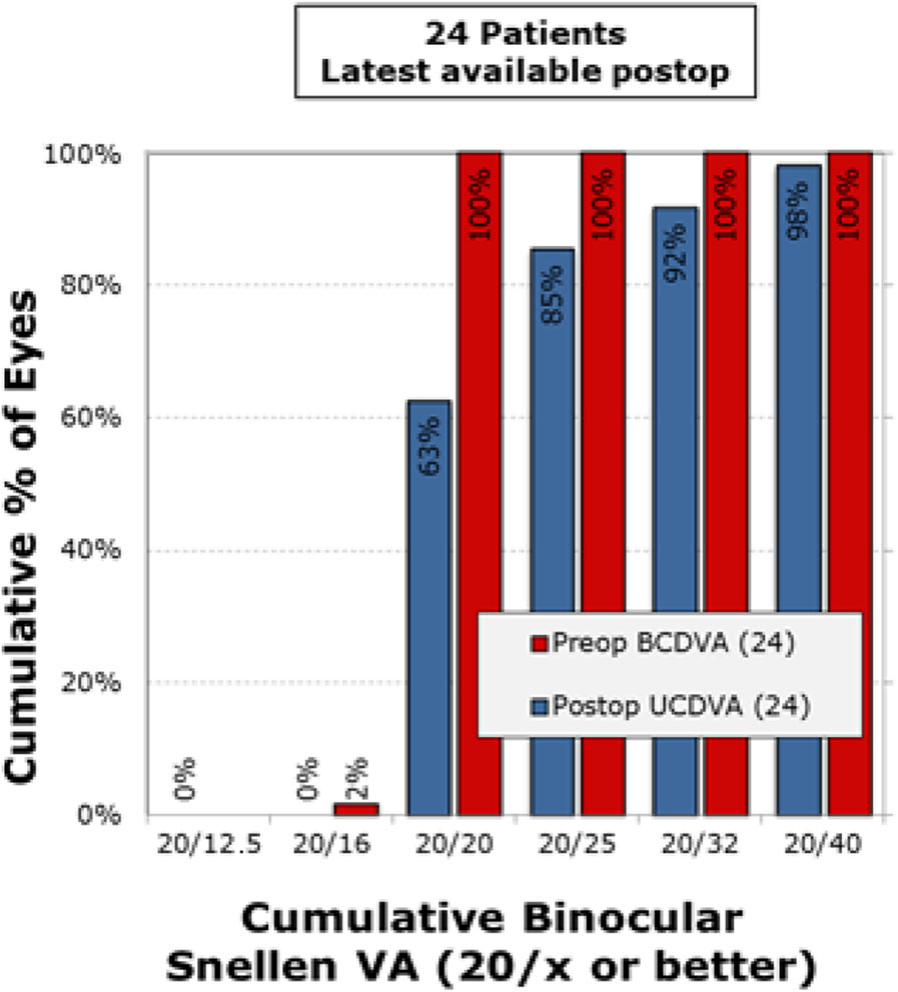
Cumulative UCDVA Binocular Snellen Visual Acuity (20/x or better)

**Fig. 2:**
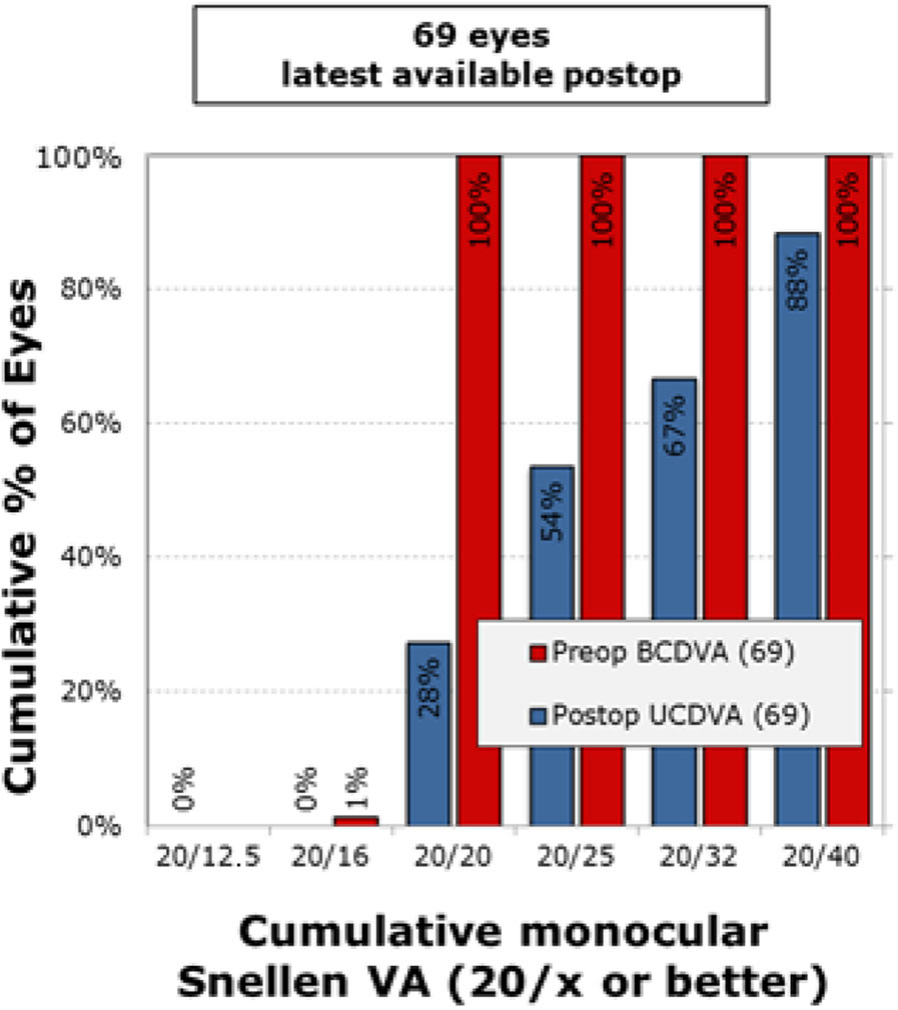
Cumulative UCDVA Monocular Snellen Visual Acuity (20/x or better)

**Fig. 3:**
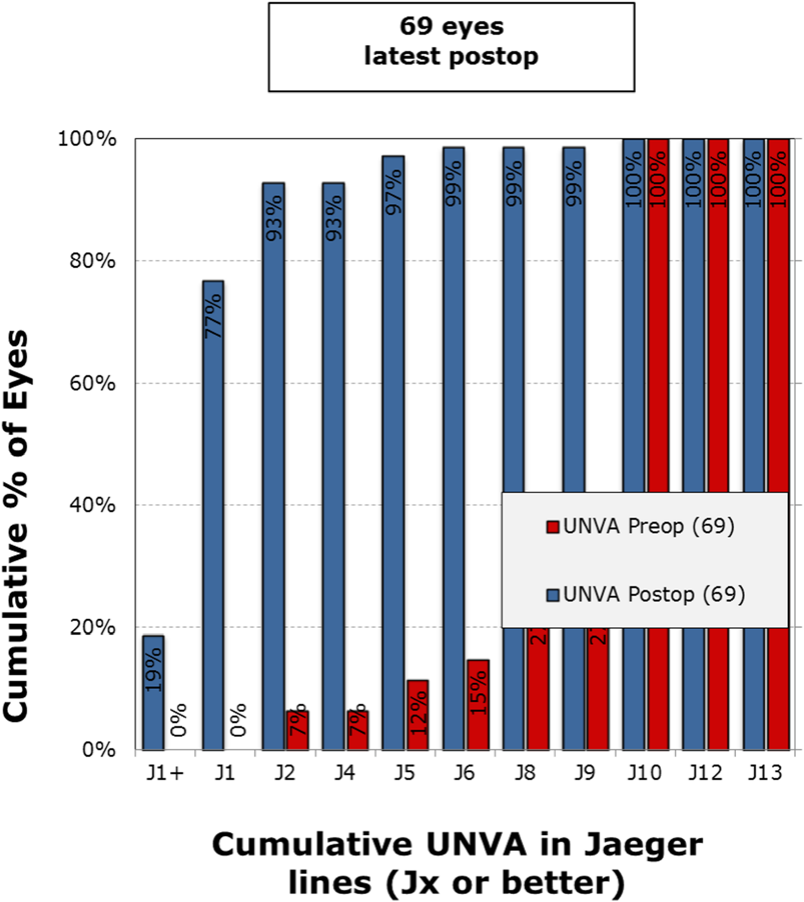
Cumulative uncorrected near visual acuity

Pre-operatively, the mean monocular UDVA was log MAR 0.35 (20/50), UIVA was 0.35 (20/50), and UNVA was 0.61 (J9). At 6 months post operatively, the binocular cumulative UDVA (Fig. 1) was 20/20 or better in 63% of patients and 20/25 or better in 85% of patients. Figure 2 shows the uncorrected cumulative monocular UDVA at 6 months wherein 54% of patients were 20/25 or better. For the uncorrected near visual acuity at 6 months post operatively, Fig. 3 shows the cumulative reading abilities wherein 93% achieved J2 or better.

### Predictability

**Fig. 4:**
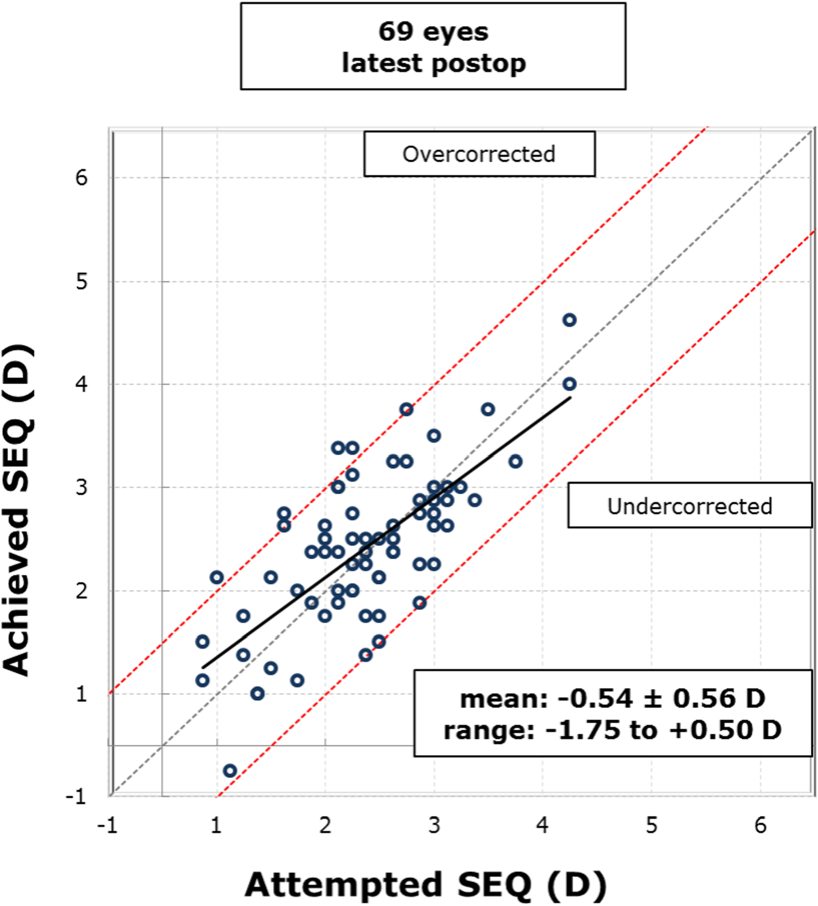
Intended spherical equivalent (SE) refraction versus the achieved SE refraction

**Fig. 5:**
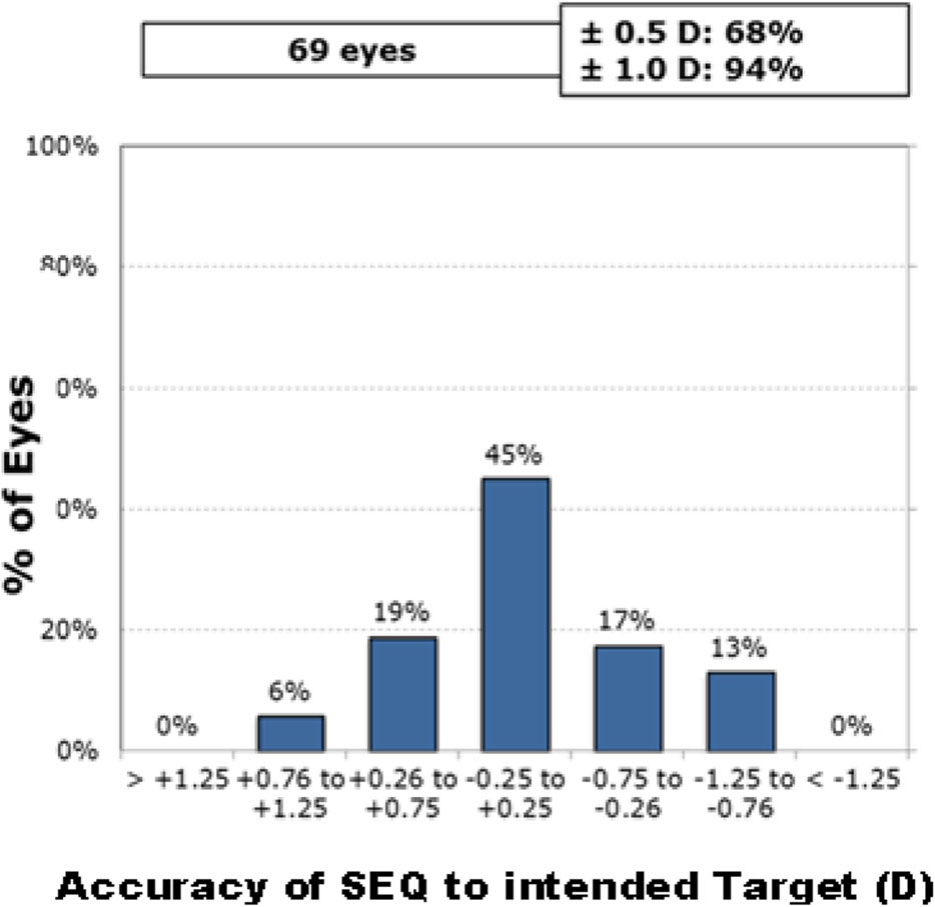
Spherical Equivalent Accuracy, Achieved vs. Target

**Fig. 6:**
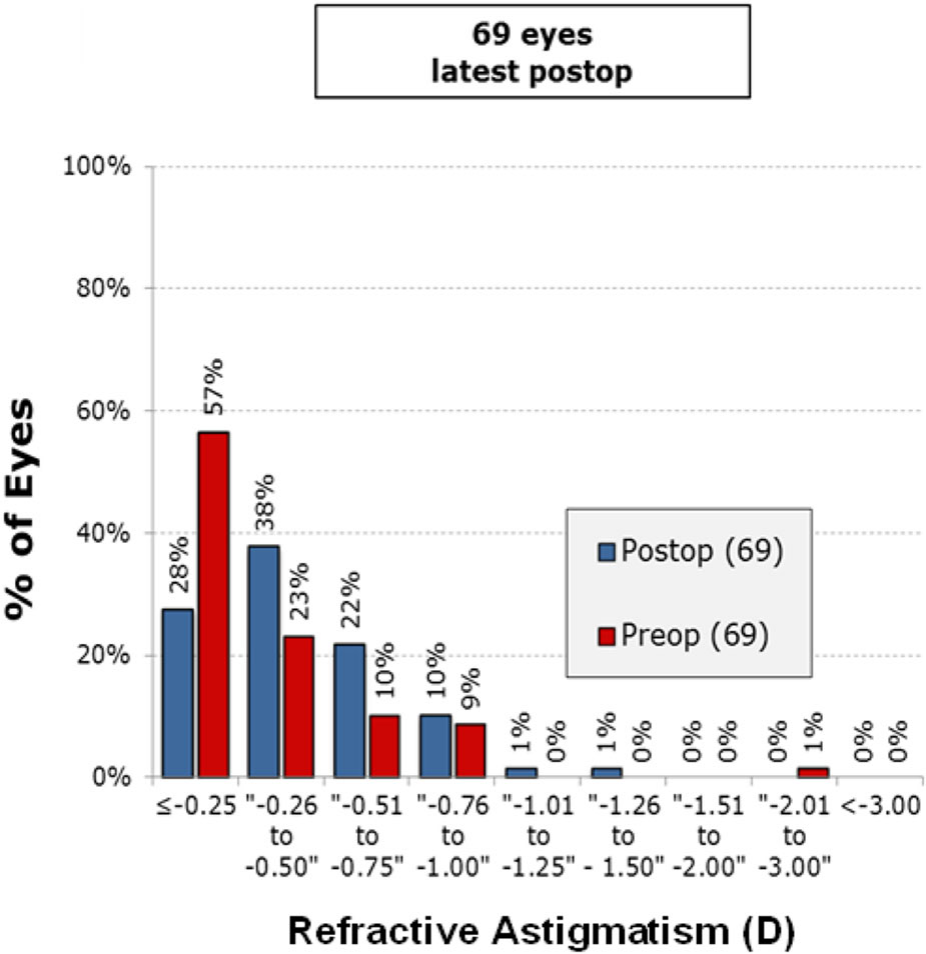
Refractive Astigmatism Amplitude

Figure 4 shows the intended SE refraction versus the achieved SE refraction at 6 months postoperatively. Figure 5 shows the SE refractive accuracy while Fig. 6 shows the postoperative refractive astigmatism amplitude.

### Safety

**Fig. 7:**
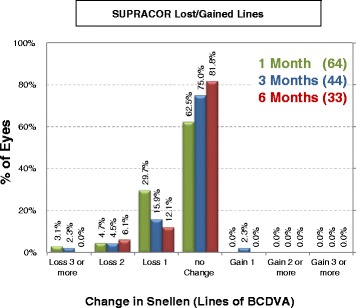
Gains/Loss of lines of all cases

**Fig. 8:**
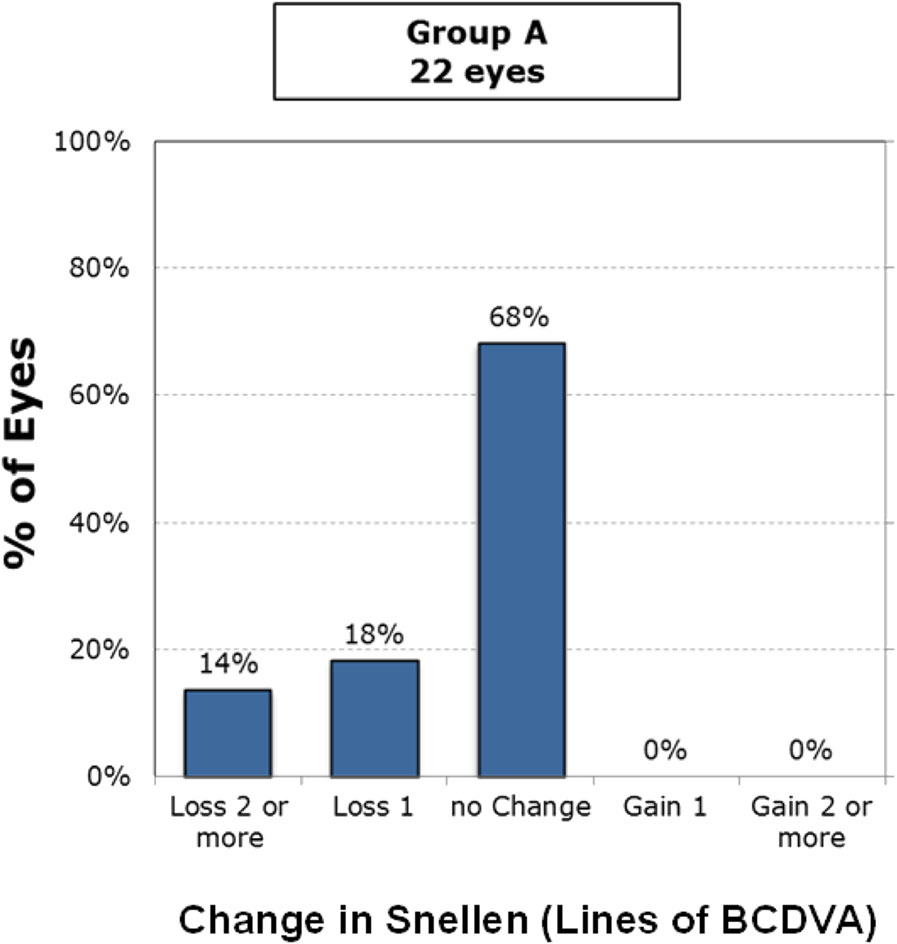
Gains/Loss of lines of Group A (Supracor + Supracor)

**Fig. 9:**
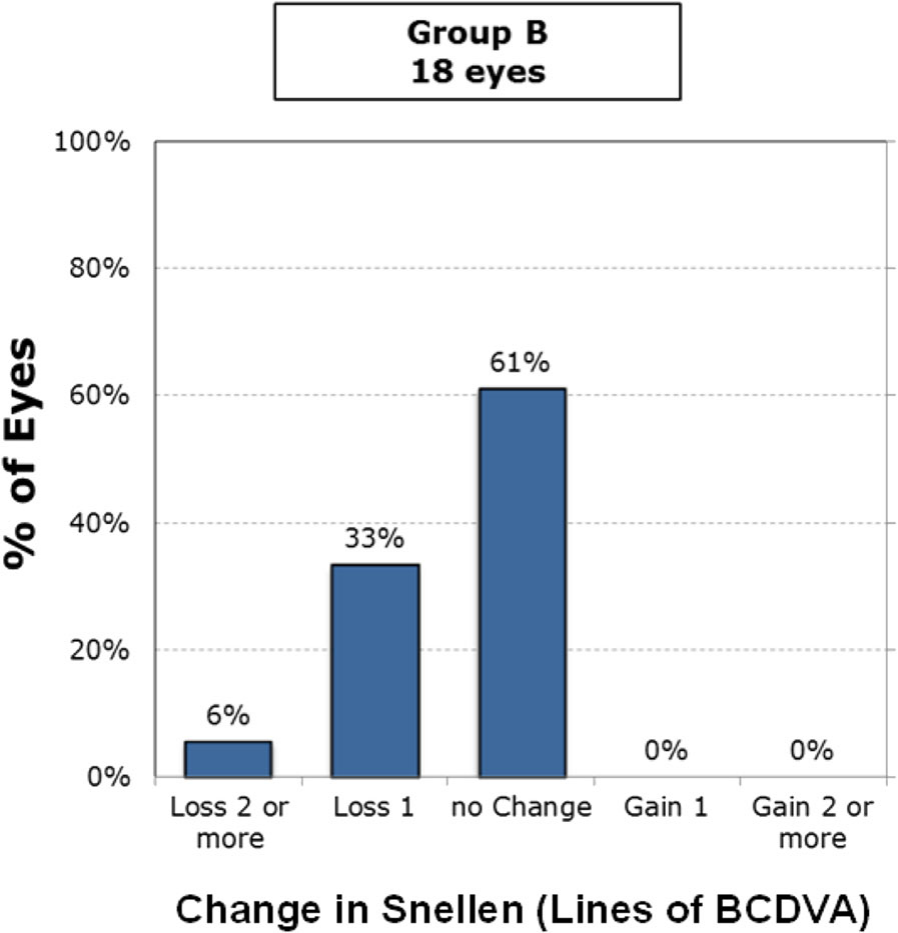
Gains/Loss of lines of Group B (Supracor + LASIK)

**Fig. 10:**
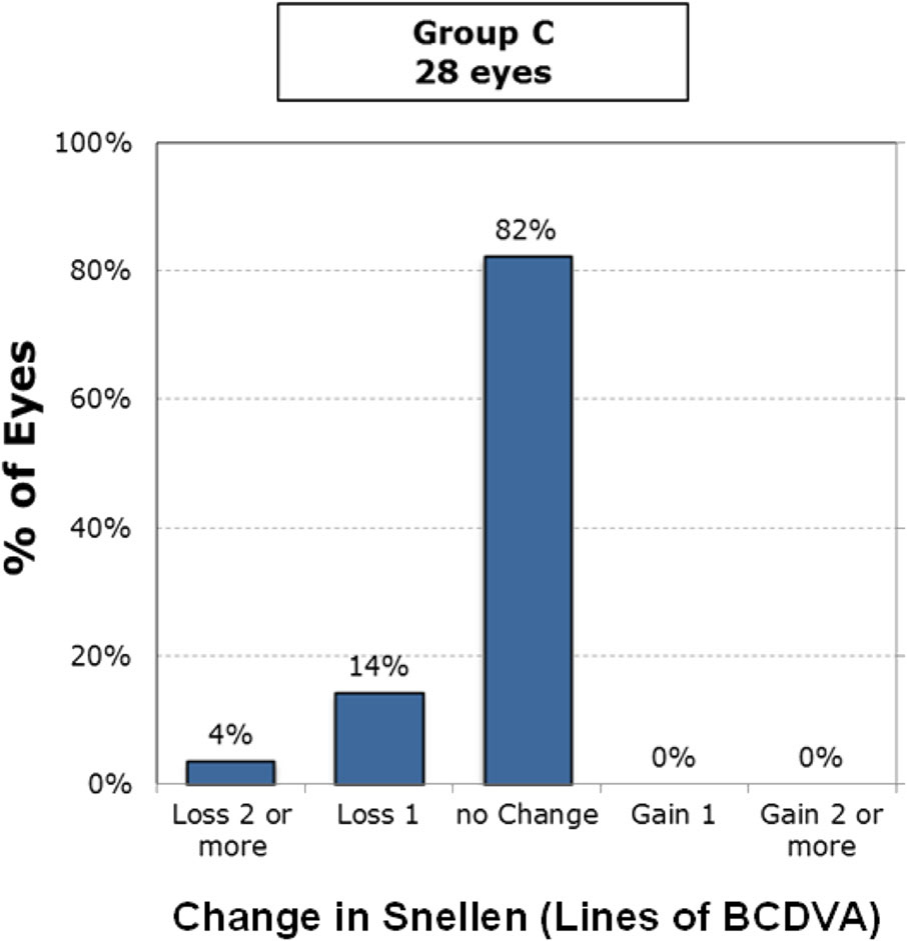
Gains/Loss of lines of Group C (Supracor + No treatment)

At 6 months postoperatively, 6% lost two lines of best-corrected distance visual acuity (BCDVA) and 12% lost one line among all Supracor eyes (Fig. 7). Among the subdivided groups, 14% of the bilateral Supracor eyes (Group A) lost two lines and 18% lost one line of BCDVA (Fig. 8), 6% of Group B lost two lines and 33% lost one line of BCDVA (Fig. 9), and 4% of Group C loss two lines and 14% lost one line of BCDVA (Fig. 10). One eye developed an epithelial ingrowth which was resolved after cleaning the flap interface. One eye developed steroid-induced ocular hypertension which resolved after stopping prednisolone and instilling brimonidine plus timolol eye drops. There were no intraoperative complications but four patients eventually needed enhancement after 2 months (*n* = 1), 6 months (*n* = 1), 12 months (*n* = 1), and 14 months (*n* = 1) follow up. No other complications were observed.

## Binocular vision results

In Group A, the mean MRSE of the Supracor-treated dominant eye was 1.81 ± 0.32 D preoperatively and −0.47 ± 0.24 D at 6 months. The non-dominant Supracor-treated eye had MRSE of 1.76 ± 0.38 D preoperatively and −0.59 ± 0.64 D after 6 months. In Group B, the mean MRSE of the dominant eye was 1.63 ± 0.46 D preoperatively and 6 months after hyperopic LASIK was 0.01 ± 0.21 D. The non-dominant Supracor-treated eye had a mean MRSE of 1.81 ± 0.72 D and was −0.55 ± 0.63 D 6 months after Supracor. In Group C, the mean MRSE of the dominant eye left untreated was 0.76 ± 0.38 D preoperatively and remained at +0.60 ± 0.46 D 6 months after surgery of the fellow eye. The mean MRSE of the non-dominant Supracor-treated eye was 0.78 ± 0.48 D preoperatively and was −0.45 ± 0.38 D 6 months after Supracor (Table 3).

**Table 3:** MRSE of Treatment Groups (D)

MRSE	Group A Dominant (Supracor)	Group A Non Dominant (Supracor)	Group B Dominant (LASIK)	Group B Non-Dominant (Supracor)	Group C Dominant (No treatment)	Group C Non-Dominant (Supracor)
Pre-Op	1.81 ± 0.32	1.76 ± 0.38	1.63 ± 0.46	1.81 ± 0.72	0.76 ± 0.38	0.78 ± 0.48
1 month	−0.39 ± 0.55	−0.51 ± 0.61	−0.16 ± 0.37	−1.06 ± 0.78	0.68 ± 0.44	−0.78 ± 0.59
3 months	−0.32 ± 0.40	−0.53 ± 0.42	0.10 ± 0.31	−0.76 ± 0.63	0.66 ± 0.39	−0.51 ± 0.60
6 months	−0.47 ± 0.24	−0.59 ± 0.64	0.01 ± 0.21	−0.55 ± 0.63	0.60 ± 0.46	−0.45 ± 0.38

Six months after treatment, the mean binocular UDVA of Group A (Table 4) was 0.02 ± 0.05 logMAR (20/20), Group B was 0.00 ± 0.00 logMAR (20/20) and Group C was 0.02 ± 0.05 logMAR (20/20). The mean binocular UIVA of Group A (Table 5) was −0.10 ± 0.08 logMAR (20/16), Group B was 0.00 ± 0.05 logMAR (20/20) and Group C was −0.10 ± 0.17 logMAR (20/16). The mean binocular UNVA of Group A (Table 6) was −0.07 ± 0.05 logMAR (J1+), Group B was 0.00 ± 0.14 logMAR (J1) and Group C was 0.00 ± 0.10 logMAR (J1). The mean binocular UDVA, UIVA, UNVA for each treatment group remained stable and showed no significant difference between groups throughout the follow up period.

**Table 4:** Binocular UDVA (logMAR)

Binocular UDVA	Group A Bilateral Supracor	Group B Supracor + Standard LASIK	Group C Supracor + Untreated	One way ANOVA (*p* value)
Pre OP	0.63 ± 0.18 (20/80)	0.43 ± 0.21 (20/50)	0.04 ± 0.06 (20/20)	0.00
1 Month	0.09 ± 0.10 (20/25)	0.03 ± 0.05 (20/20)	0.04 ± 0.05 (20/20)	0.12
3 Months	0.01 ± 0.00 (20/20)	0.03 ± 0.06 (20/20)	0.07 ± 0.06 (20/20)	0.15
6 Months	0.02 ± 0.05 (20/20)	0.00 ± 0.00 (20/20)	0.02 ± 0.05 (20/20)	0.36

**Table 5:** Binocular UIVA (logMAR)

Binocular UIVA	Group A Bilateral Supracor	Group B Supracor + Standard LASIK	Group C Supracor + Untreated	One way ANOVA (*p* value)
Pre OP	0.40 ± 0.00 (20/50)	0.40 ± 0.00 (20/50)	0.30 ± 0.11 (20/40)	0.00
1 Month	−0.02 ± 0.09 (20/20)	−0.01 ± 0.07 (20/20)	0.05 ± 0.06 (20/20)	0.053
3 Months	−0.05 ± 0.07 (20/20)	0.04 ± 0.10 (20/20)	0.00 ± 0.11 (20/20)	0.13
6 Months	−0.10 ± 0.08 (20/16)	0.00 ± 0.05 (20/20)	−0.10 ± 0.17 (20/16)	0.19

**Table 6:** Binocular UNVA (logMAR)

Binocular UNVA	Group A Bilateral Supracor	Group B Supracor + Standard LASIK	Group C Supracor + Untreated	One way ANOVA (*p* value)
Pre OP	0.70 ± 0.00 (J10)	0.68 ± 0.06 (J10)	0.52 ± 0.21 (J8)	0.01
1 Month	−0.02 ± 0.06 (J1)	−0.04 ± 0.07 (J1)	−0.02 ± 0.07 (J1)	0.66
3 Months	−0.01 ± 0.07 (J1)	−0.03 ± 0.10 (J1)	−0.02 ± 0.04 (J1)	0.85
6 Months	−0.07 ± 0.05 (J1+)	0.00 ± 0.14 (J1)	0.00 ± 0.10 (J1)	0.56

### Corneal Topography

**Fig. 11:**
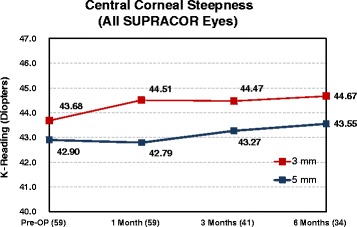
Post-operative corneal steepening

Corneal curvature tests were performed on all Supracor-treated eyes using the Orbscan IIz Corneal Topography (Bausch & Lomb, Munich, Germany). Comparing preoperative and 6 month postoperative measurements, a mean 1.0 D corneal steeping at the central 3 mm and 0.7 D change at the central 5 mm were observed (Fig. 11).

**Fig. 12:**
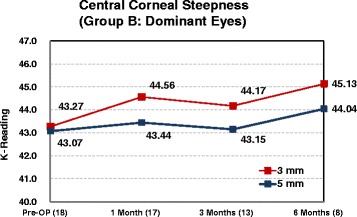
Corneal Steepness (Group B)

**Fig. 13:**
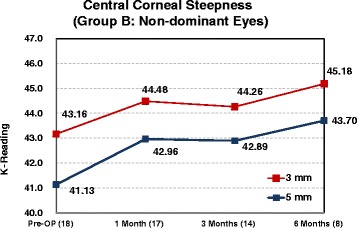
Supracor treated eyes corneal steepness

Corneal curvature changes from preoperative to the 6 month postoperative visit were compared between Supracor-treated eyes and hyperopic LASIK-treated eyes in Group B patients (Fig. 12). Mean steepening at 3 mm was similar between treatments while the degree of steepening at 5 mm was observed more in the Supracor-treated eyes (Fig. 13).

### Higher order aberrations

**Fig. 14:**
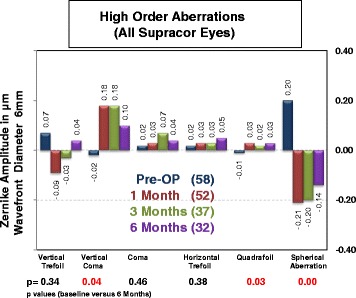
Higher order aberrations of all Supracor treated eyes

Higher order aberrations were measured using the Zywave II wavefront Aberrometry (Bausch & Lomb, Munich, Germany). Vertical coma increased from −0.02 μm preoperatively to +0.10 μm at 6 months after Supracor (*p* = 0.04). Quadrafoil increased from −0.01 μm preoperatively to +0.03 μm at 6 months (*p* = 0.03). Fourth order spherical aberration changed from +0.20 μm preoperatively to −0.14 μm at 6 months postoperatively (*p* < 0.0001). Horizontal coma, vertical trefoil, and horizontal trefoil did not change significantly (Fig. 14).

**Fig. 15:**
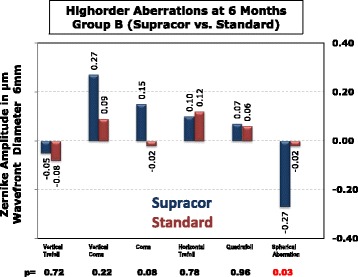
Higher order aberrations of Group B

The change in higher order aberrations was compared between Supracor-treated and hyperopic LASIK-treated eyes in Group B patients. The increase in negative 4^th^order spherical aberration (*p* = 0.03) was higher in the Supracor-treated eyes compared to eyes that underwent hyperopic Lasik. No significant difference was seen on vertical trefoil, horizontal trefoil, vertical coma, horizontal coma, and quadrafoil (Fig. 15).

### Retreatments

Out of the 69 hyperopic eyes that had Supracor, four eyes underwent retreatment (5.7%). The indications for retreatment were decreased UNVA and a regression towards hyperopia of the refractive spherical equivalent of their post-Supracor eyes. A non-wavefront standard LASIK algorithm was used with a target of −0.50 D. We noted an improvement in the UNVA for all of the patients post-enhancement. Their post-enhancement results are shown below (Table 7).

**Table 7:** Post-Enhancement Visual and Refractive Outcomes

Pre Supracor	Patient A	Patient B	Patient C	Patient D
UDVA	20/20	20/30	20/60	20/25
UIVA	20/32	20/50	20/50	20/25
UNVA	J5	J10	J10	J10
Sphere (D)	+0.50	+1.25	+1.75	+0.50
Cylinder (D)	−0.25	−0.50	−0.75	−0.75
Spherical Equivalent (D)	+0.375	+1.00	+1.375	+0.125
Pre Enhancement	14 months	6 months	1 year	2 months
UDVA	20/20	20/25	20/20	20/25
UIVA	20/25	20/16	20/40	20/25
UNVA	J3	J1 (doubling)	J10	J5
Sphere (D)	Plano	Plano	+0.50	+0.50
Cylinder (D)	−0.25	−0.50	0.00	−0.25
Spherical Equivalent (D)	−0.125	−0.25	+0.50	+0.375
Latest follow up	2.5 years	1 year	3 weeks	6 months
UDVA	20/20	20/30	20/50	20/30
UIVA	20/25	20/25	20/30	20/25
UNVA	J1	J1	J2	J1
Sphere (D)	−0.25	−1.00	−0.50	−0.50
Cylinder (D)	−0.25	−0.50	−0.25	−1.00
Spherical Equivalent (D)	−0.325	−1.25	−0.63	−1.00

*UDVA*= uncorrected distance visual acuity; *UIVA*= uncorrected intermediate visual acuity; *UNVA*= uncorrected near visual acuity

## Discussion

Surgical correction for presbyopia remains one of the most challenging aspects of refractive surgery. Several LASIK-based strategies have been or are being developed to address this gap. Our retrospective study presents our early experience and outcomes with the Supracor algorithm (Bausch and Lomb Technolas, Munich, Germany) on hyperopic presbyopic patients. In addition to reporting the efficacy and safety of the algorithm, we analyzed the refractive and binocular visual outcomes of three subgroups of patients–bilateral Supracor (Group A), one eye Supracor with fellow eye hyperopic LASIK (Group B) and one eye Supracor with fellow eye untreated (Group C).

**Fig. 16:**
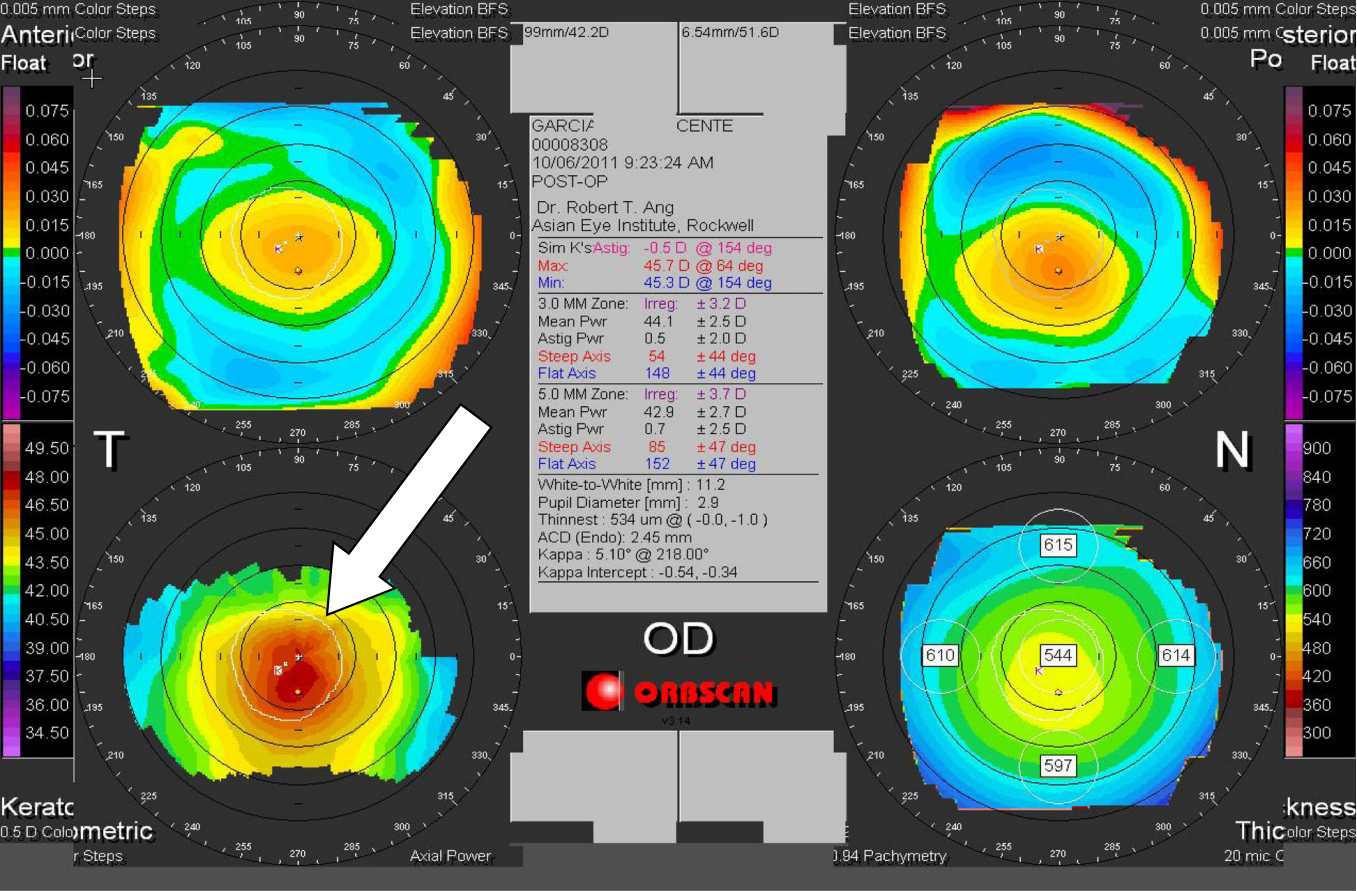
Post-Operative Zywave Aberration Sample Image

The Supracor algorithm creates an elevation 12 μm high and 3 mm in diameter in the central cornea along the visual axis. The elevation is surrounded by an aspheric optimized area where a smooth transition creates the intermediate vision zone while the distance vision zone is positioned in the periphery (Fig. 16). This varifocal principle allows simultaneous good distance, intermediate and near vision. Supracor makes use of the central-near, peripheral-distance concept wherein during natural accommodation when the eye focuses on near objects, the pupil constricts and the eye looks thru the near-add elevation. When the eye is looking at a distance, the pupil dilates and allows the peripheral rays to pass through the aspheric optimized periphery to improve distance vision. Supracor provides approximately two dioptres of near vision. With the refractive target of −0.50 D spherical equivalent, it is believed that this −0.50 D [13] residual refractive error does not significantly sacrifice distance vision but adds to the 2.0 D near add, thereby increasing the ability to read small print with a total add power of 2.5 D.

In our study, the Supracor LASIK treatment was able to achieve a mean spherical equivalent of −0.43 ± 0.59 D at the 6 month follow up (Table 2). This resulted in an 85% uncorrected distance vision of 20/25 (Fig. 1), and 93% near vision of J2 (Fig. 3). A noticeable trend was that the eyes were initially more myopic but settled near the target refraction over time. This refractive trend mirrors the visual recovery wherein distance vision was initially unclear but improved over time while the near and intermediate vision was good from the start.

Safety is a significant concern for new treatments. In our study, 7% lost two or more lines of BCDVA at 6 month post-operative period. We attribute the lost lines primarily to dry eye, which affects older presbyopic patients and is aggravated by the LASIK procedure itself. Therefore, we placed punctual plugs and start patients on cyclosporine (Restasis, Allergan, USA). Another possible reason for lost lines of vision is the induced higher order aberrations. Similar and even greater losses of BCDVA have been reported in various cornea-based presbyopia treatments. Alió et al. [4] reported a loss of a maximum of two lines of BCDVA in 28% of cases in their study of central presbyopic LASIK in hyperopic patients. A study by Ryan et al. [13] showed a similar rate of loss of two or more lines of monocular BCDVA at 6.5%. Using the biaspheric ablation profile of PresbyMax (Schwind, Kleinostheim, Germany), Uthoff et al. [14] found that 15% lost two lines or more of BCDVA monocularly and 13% binocularly at 6 months in the treatment of presbyopic hyperopic, emmetropic, and myopic patients while Cosar and Sener [15] reported a loss of 28% in one line and 10% loss of two lines of BCDVA at 6 months.

Ryan and Keefe [13] reported that Supracor provided a high level of spectacle independence for near vision, but around 22% had unsatisfactory uncorrected distance vision that required retreatment. In our case series, 5.7% of eyes eventually needed retreatment. However, unlike the Ryan and Keefe study wherein the main indication for retreatment was poor uncorrected distance vision, our indication for all four enhancements was deterioration of uncorrected near vision.

We had three subgroups of patients who underwent Supracor because we customized our treatment recommendations based on individualized vision conditions. Hyperopic patients seeking presbyopia treatment can have minimal hyperopia wherein distance vision is good even without glasses or visually significant hyperopia wherein distance vision is poor without eyeglasses. Checking eye dominance is paramount during the screening process because the non-dominant eye gets a Supracor treatment. For patients with uncorrected distance vision of 20/30 or better in the dominant eye, we would suggest Supracor treatment only in the non-dominant eye and no treatment in the fellow eye. If the dominant eye has an uncorrected distance vision worse than 20/30, we give the patient the option of hyperopic LASIK in the dominant eye if they need to ensure good far vision or Supracor LASIK in the dominant eye if they prefer good near vision with the understanding that there is a mild sacrifice in distance vision. Our results support this treatment differentiation because the mean uncorrected distance vision of Supracor-treated eyes is 20/25 and not 20/20 which is the typical outcome in hyperopic LASIK-treated eyes. In addition, because of the initial overly myopic outcomes, improvement in distance vision takes time so it is important to set the proper expectations if patients decide on bilateral Supracor Lasik. Analyzing the three subgroups yielded similarly good uncorrected distance, intermediate and near vision binocularly. This finding suggests that the differences between monolateral and bilateral Supracor are minimal but more importantly, these outcomes confirm that Supracor LASIK is a viable option for presbyopia treatment.

Corneal steepening occurs after hyperopic LASIK but to a greater magnitude after Supracor treatments (Figs 11 and 12). Vertical coma, quadrafoil, and negative spherical aberration were shown to have significantly increased after Supracor. This was consistent with previous studies reporting that LASIK increases higher order wavefront aberrations of the cornea, dependent on the amount of refractive correction; [16] and that hyperopic LASIK induced negative spherical aberrations and more third- and fifth-order coma-like aberrations than myopic LASIK [17]. Taken together, the mechanism of action of Supracor can be better understood. The central elevation and aspheric optimized mid periphery created by the Supracor procedure is manifested as central corneal steepening and a negative spherical aberration that are believed to improve depth of focus. Coupled with a −0.50 D refractive outcome, presbyopia treatment is enhanced.

## Conclusions

In conclusion, our study has shown that the Supracor algorithm is safe and effective for hyperopic patients in correcting refractive error and presbyopia simultaneously in a single LASIK treatment.  Supracor can be used in one or both eyes depending on patient needs and expectations.  Though treatment modalities in each subgroup translated to significantly good outcomes, we advocate a conservative approach of monolateral Supracor treatment to ensure good distance vision while improving near vision in a modified mini-monovision approach.  Enhancements can be performed to further improve distance or near vision outcomes depending on patients' needs or occurrence of regression.  The limitations of our study are that monolateral or bilateral treatments were not randomly assigned in a prospective manner, the study population is quite small, questionnaires on patient satisfaction and spectacle independence were not provided to patients for analysis, and quality of vision tests were performed.  As a clinician, we recommend careful patient selection, managing, expectations and critical decision-making to increase the satisfaction rate of patients seeking any form of presbyopia LASIK treatment such as Supracor.

## Abbreviations

BCDVA: Best corrected visual acuity
DCNVA: Distance corrected near visual acuity
LogMAR: Logarithm of minimum angle of resolution
MR: Manifest refraction
MRSE: Manifest refract spherical equivalent
UDVA: Uncorrected distance visual acuity
UIVA: Uncorrected intermediate visual acuity
UNVA: Uncorrected near visual acuity
